# Supplementary material for: Deep learning empowered sensor fusion boosts infant movement classification
Source: Commun Med (Lond). 2025 Jan 14;5:16. doi: 10.1038/s43856-024-00701-w (PMC11733215; doi:10.1038/s43856-024-00701-w)
Supplement: Supplementary file 2 — Supplementary Information [file 43856_2024_701_MOESM2_ESM.pdf]

Deep learning empowered sensor fusion boosts infant movement classification

Tomas Kulvicius[1,2,3,12,*], Dajie Zhang[2,4,12], Luise Poustka[2], Sven Bölte[5,6,7], Lennart Jahn[1,3], Sarah Flügge[1], Marc Kraft[8], Markus Zweckstetter[2,9,10], Karin Nielsen-Saines[11], Florentin Wörgötter[3,13], and Peter B Marschik[1,2,4,5,13,*]

[1]Child and Adolescent Psychiatry and Psychotherapy, University Medical Center Göttingen, Leibniz ScienceCampus Primate Cognition and German Center for Child and Adolescent Health (DZKJ), Göttingen, Germany

[2]Department of Child and Adolescent Psychiatry, University Hospital Heidelberg, Heidelberg University, Heidelberg, Germany

[3]Department for Computational Neuroscience, Third Institute of Physics - Biophysics, Georg-August-University of Göttingen, Göttingen, Germany

[4]iDN – interdisciplinary Developmental Neuroscience, Division of Phoniatrics, Medical University of Graz, Graz, Austria

[5]Center of Neurodevelopmental Disorders (KIND), Department of Women's and Children's Health, Center for Psychiatry Research, Karolinska Institutet & Region Stockholm, Stockholm, Sweden

[6]Child and Adolescent Psychiatry, Stockholm Health Care Services, Region Stockholm, Stockholm, Sweden

[7]Curtin Autism Research Group, Curtin School of Allied Health, Curtin University, Perth, Australia

[8]Department of Medical Engineering, Technical University Berlin, Berlin, Germany

[9]German Center for Neurodegenerative Diseases (DZNE), Göttingen, Germany

[10]Department for NMR-based Structural Biology, Max Planck Institute for Multidisciplinary Sciences, Göttingen, Germany

[11]Department of Pediatrics, David Geffen UCLA School of Medicine, Los Angeles, CA, USA

[12]These authors contributed equally

[13]These authors jointly supervised this work

[*]Corresponding authors: Dr. Tomas Kulvicius, tomas.kulvicius@med.uni-goettingen.de; Prof. Peter B Marschik, peter.marschik@med.uni-heidelberg.de

Supplementary information

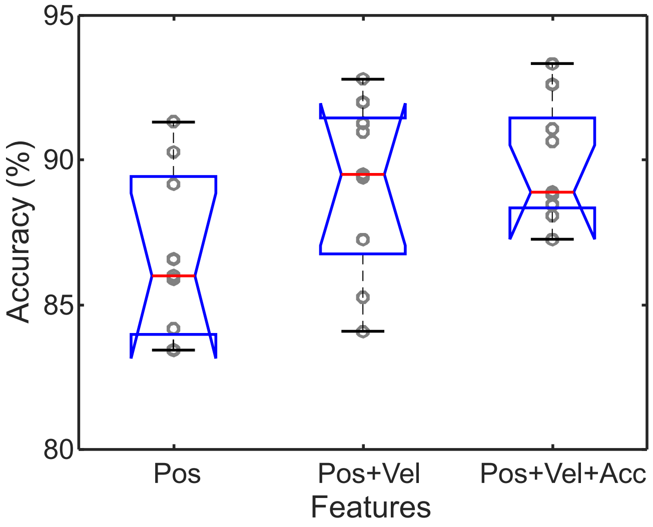

Supplementary Figure 1: Comparison of the classification accuracies on the test sets (9-fold cross-validation) using different skeleton features ($n = 9$). Only positions of the key points (Pos), positions and velocities of the key points (Pos+Vel), and positions, velocities and accelerations of the key points (Pos+Vel+Acc). We used CNN architecture with three convolutional layers (kernel parameters for each layer [numbers of kernels, filter size]: 8, 13x1; 32, 17x1; 64, 25x1) and one fully connected layer (256 units). Gray circles correspond to the classification accuracies for each fold. The box lines correspond to the lower quartile, median, and upper quartile values, and the whiskers represent ranges of the rest of the data. The notches represent a robust estimate of the uncertainty about the medians.

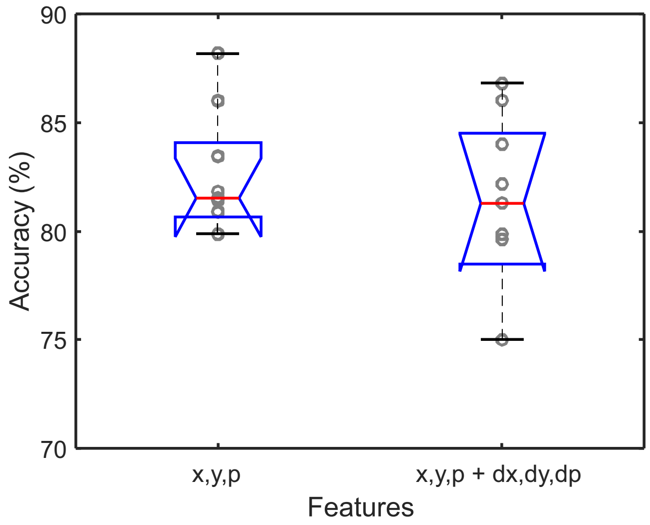

Supplementary Figure 2: Comparison of the classification accuracies on the test sets (9-fold cross-validation) using different pressure mat features ($n = 9$). Only x, y, p features, and x, y, p features and their first derivatives dx, dy, dp. We used CNN architecture with three convolutional layers (kernel parameters for each layer [numbers of kernels, filter size]: 8, 13x1; 32, 17x1; 64, 25x1) and one fully connected layer (256 units). Gray circles correspond to the classification accuracies for each fold. The box lines correspond to the lower quartile, median, and upper quartile values, and the whiskers represent ranges of the rest of the data. The notches represent a robust estimate of the uncertainty about the medians.

Supplementary Table 1: Parameter values for the hyperparamter tuning of the convolutional neural network architectures.

	Bayesian optimisation	Grid search (fine tuning)
Number of conv. layers	{1, 2, 3}	3
Number of kernels	Conv 1-3: {4, 8, 16, 32, 64, 128}	Conv 1: {4, 8}<br>Conv 2: {8, 16, 32, 64}<br>Conv 3: {8, 16, 32, 64}
Kernel size	Conv 1-3: {5×1, 7×1, 9×1, 13×1, 17×1, 25×1, 33×1}	Conv 1: {13×1, 17×1, 25×1}<br>Conv 2: {7×1, 9×1, 13×1, 17×1, 25×1}<br>Conv 3: {9×1, 13×1, 17×1, 25×1, 33×1}
Number of fully connect. (FC) layers	{1, 2}	1
Number of FC units	{64, 128, 256}	{128, 256}

Supplementary Table 2: Hyperparameters of the network architectures for different sensor modalities.

Model #	Input dim.	Convolutional layers (# of kernels, kernel size)			FC layer (# of units)	Out. dim.
		Conv 1	Conv 2	Conv 3	FC 1	
Pressure mat (MAT)						
1	500×6	8, 13×1	64, 17×1	16, 25×1	256	1
2		8, 13×1	32, 7×1	16, 33×1	128	
3		4, 17×1	64, 17×1	16, 13×1	128	
4		4, 17×1	16, 13×1	8, 33×1	128	
5		4, 17×1	64, 25×1	64, 17×1	256	
6		4, 17×1	32, 17×1	8, 13×1	128	
7		4, 17×1	8, 7×1	8, 33×1	128	
8		4, 13×1	16, 9×1	64, 33×1	256	
9		4, 25×1	32, 13×1	16, 13×1	256	
10		4, 17×1	64, 7×1	16, 9×1	128	
Inertial measurement units (IMU)						
1	300×36	8, 25×1	8, 17×1	64, 25×1	256	1
2		8, 25×1	64, 13×1	8, 25×1	256	
3		4, 13×1	64, 17×1	32, 33×1	128	
4		8, 17×1	8, 17×1	8, 17×1	128	
5		4, 17×1	64, 13×1	32, 33×1	128	
6		8, 25×1	32, 17×1	64, 33×1	128	
7		4, 25×1	32, 9×1	8, 9×1	128	
8		8, 25×1	8, 25×1	8, 33×1	128	
9		8, 25×1	32, 25×1	8, 33×1	128	
10		8, 17×1	64, 25×1	8, 13×1	128	
Video [skeleton] (VID)						
1	250×60	4, 13×1	32, 25×1	16, 25×1	128	1
2		4, 13×1	32, 17×1	64, 9×1	256	
3		8, 25×1	8, 13×1	64, 33×1	128	
4		4, 25×1	64, 7×1	32, 17×1	128	
5		4, 25×1	32, 25×1	16, 13×1	256	
6		4, 25×1	8, 25×1	64, 25×1	256	
7		4, 13×1	8, 7×1	64, 13×1	128	
8		8, 13×1	32, 13×1	64, 25×1	256	
9		8, 13×1	32, 7×1	64, 9×1	256	
10		4, 13×1	16, 25×1	64, 13×1	128	

Supplementary Table 3: Hyperparameters of the network architectures for the sensor fusion.

Model #	Input dim.	Convolutional layers (# of kernels, kernel size)			FC layer (# of units)	Out. dim.
		Conv 1	Conv 2	Conv 3	FC 1	
Sensor fusion (MAT+IMU+VID)						
1	250×102	4, 25×1	16, 7×1	64, 33×1	128	1
2		8, 13×1	8, 7×1	16, 25×1	256	
3		4, 25×1	32, 7×1	64, 33×1	256	
4		4, 13×1	16, 7×1	64, 17×1	128	
5		8, 17×1	64, 17×1	16, 17×1	256	
6		8, 25×1	8, 13×1	16, 13×1	256	
7		8, 17×1	8, 9×1	8, 9×1	256	
8		4, 17×1	64, 9×1	64, 25×1	256	
9		4, 13×1	16, 9×1	32, 9×1	256	
10		8, 17×1	8, 9×1	8, 17×1	256	

Supplementary Table 4: Classification results for the models trained on different sensor modalities. Average classification measures obtained from the 9-fold cross-validation together with confidence intervals of mean (CI 95%) are shown for each case ($n = 9$). Models' hyperparameters are specified in Supplementary Table 2.

Model #	Sens. (%) [CI]	Spec. (%) [CI]	BA (%) [CI]
Pressure mat (MAT)			
1	86.17 [82.78 89.55]	77.95 [69.68 86.22]	**82.06** [77.11 87.00]
2	86.84 [83.63 90.04]	74.71 [66.07 83.35]	80.77 [75.85 85.69]
3	84.77 [80.73 88.80]	76.28 [67.87 84.69]	80.53 [76.50 84.55]
4	86.18 [82.70 89.65]	73.95 [65.28 82.62]	80.06 [74.84 85.29]
5	86.10 [81.95 90.25]	72.75 [64.18 81.32]	79.43 [74.76 84.09]
6	86.05 [81.48 90.62]	72.28 [64.18 80.38]	79.17 [75.75 82.58]
7	86.64 [81.42 91.86]	71.10 [59.84 82.35]	78.87 [73.21 84.52]
8	87.03 [82.85 91.21]	69.41 [59.65 79.17]	78.22 [73.19 83.24]
9	85.54 [82.30 88.79]	69.59 [56.95 82.23]	77.57 [70.89 84.24]
10	87.91 [85.07 90.75]	66.88 [54.93 78.83]	77.39 [71.15 83.64]
Inertial measurement units (IMU)			
1	92.91 [90.28 95.55]	87.52 [83.47 91.57]	**90.22** [87.61 92.82]
2	93.30 [89.96 96.64]	86.75 [81.38 92.12]	90.02 [87.18 92.87]
3	94.92 [92.51 97.33]	85.03 [80.30 89.75]	89.97 [87.47 92.47]
4	91.48 [88.95 94.02]	87.70 [81.30 94.10]	89.59 [86.06 93.12]
5	91.86 [89.15 94.56]	86.06 [77.25 94.86]	88.96 [84.94 92.97]
6	91.11 [85.72 96.49]	85.75 [77.64 93.85]	88.43 [83.63 93.22]
7	91.53 [88.54 94.53]	85.30 [81.22 89.39]	88.42 [85.35 91.49]
8	91.18 [86.56 95.79]	85.49 [78.11 92.86]	88.33 [84.60 92.06]
9	90.80 [85.35 96.25]	84.78 [79.82 89.74]	87.79 [84.43 91.15]
10	90.62 [86.98 94.26]	84.45 [79.33 89.57]	87.54 [84.20 90.87]
Video [skeleton] (VID)			
1	91.67 [89.80 93.55]	89.65 [85.89 93.41]	**90.66** [88.91 92.41]
2	90.57 [87.33 93.81]	90.45 [85.23 95.68]	90.51 [88.02 93.00]
3	90.97 [88.72 93.23]	87.90 [83.88 91.92]	89.44 [87.22 91.65]
4	92.57 [90.85 94.28]	86.15 [81.28 91.02]	89.36 [87.33 91.38]
5	92.35 [89.93 94.77]	84.80 [80.01 89.58]	88.57 [86.42 90.73]
6	89.17 [86.06 92.28]	87.40 [82.70 92.09]	88.28 [86.23 90.34]
7	90.47 [89.11 91.84]	85.97 [79.62 92.33]	88.22 [85.42 91.02]
8	91.03 [88.89 93.17]	85.20 [78.90 91.50]	88.12 [85.09 91.15]
9	90.15 [87.70 92.61]	86.07 [79.60 92.55]	88.11 [85.65 90.58]
10	91.09 [88.30 93.88]	84.53 [77.72 91.35]	87.81 [85.25 90.37]

Sens. – Sensitivity, Spec. – Specificity, BA – Balanced accuracy.
Numbers in bold font correspond to the highest classification accuracy for the specific sensor modality.

Supplementary Table 5: Classification results for the models trained on the combination of all sensor modalities. Average classification measures obtained from the 9-fold cross-validation together with confidence intervals of mean (CI 95%) are shown for each case ($n = 9$). Model parameters are specified in Supplementary Table 3.

Model #	Sens. (%) [CI]	Spec. (%) [CI]	BA (%) [CI]
Sensor fusion (MAT+IMU+VID)			
1	92.87 [88.45 97.29]	93.60 [91.01 96.19]	**93.24** [91.15 95.32]
2	94.32 [90.81 97.84]	90.39 [86.20 94.57]	92.36 [90.92 93.80]
3	92.87 [88.67 97.06]	90.78 [86.14 95.41]	91.82 [89.55 94.09]
4	94.22 [91.45 96.98]	89.40 [84.45 94.36]	91.81 [89.62 94.00]
5	93.03 [89.12 96.93]	90.02 [85.31 94.73]	91.52 [89.46 93.59]
6	93.31 [89.16 97.46]	89.26 [82.29 96.22]	91.28 [88.10 94.47]
7	91.84 [88.59 95.09]	90.52 [84.96 96.07]	91.18 [88.41 93.95]
8	92.48 [88.95 96.01]	86.92 [79.33 94.51]	89.70 [85.89 93.50]
9	91.86 [88.28 95.43]	89.94 [84.70 95.18]	90.90 [87.73 94.06]
10	91.61 [87.09 96.12]	89.47 [83.52 95.42]	90.54 [87.62 93.45]

Sens. – Sensitivity, Spec. – Specificity, BA – Balanced accuracy.

The number in bold font corresponds to the highest classification accuracy.
