# Supplementary material for: Deep learning empowered sensor fusion boosts infant movement classification
Source: Commun Med (Lond). 2025 Jan 14;5:16. doi: 10.1038/s43856-024-00701-w (PMC11733215; doi:10.1038/s43856-024-00701-w)
Supplement: Supplementary file 3 — Description of Additional Supplementary Files [file 43856_2024_701_MOESM3_ESM.pdf]

Description of Additional Supplementary Files

File name- Supplementary Data 1

File description- The source data underlying results presented in Figure 4.

File name- Supplementary Data 2

File description- The source data underlying results presented in Supplementary Figure 1 and Supplementary Figure 2.
