# Supplementary material for: Deep learning empowered sensor fusion boosts infant movement classification
Source: Commun Med (Lond). 2025 Jan 14;5:16. doi: 10.1038/s43856-024-00701-w (PMC11733215; doi:10.1038/s43856-024-00701-w)
Supplement: Supplementary file 6 — REPORTING SUMMARY [file 43856_2024_701_MOESM6_ESM.pdf]

nature portfolio

Corresponding author(s): Tomas Kulvicius, and Peter B Marschik

Last updated by author(s): Nov 4, 2024

Reporting Summary

Nature Portfolio wishes to improve the reproducibility of the work that we publish. This form provides structure for consistency and transparency in reporting. For further information on Nature Portfolio policies, see our Editorial Policies and the Editorial Policy Checklist.

Statistics

For all statistical analyses, confirm that the following items are present in the figure legend, table legend, main text, or Methods section.

n/a	Confirmed	
☐	☒	The exact sample size (*n*) for each experimental group/condition, given as a discrete number and unit of measurement
☒	☐	A statement on whether measurements were taken from distinct samples or whether the same sample was measured repeatedly
☐	☒	The statistical test(s) used AND whether they are one- or two-sided<br>*Only common tests should be described solely by name; describe more complex techniques in the Methods section.*
☒	☐	A description of all covariates tested
☒	☐	A description of any assumptions or corrections, such as tests of normality and adjustment for multiple comparisons
☐	☒	A full description of the statistical parameters including central tendency (e.g. means) or other basic estimates (e.g. regression coefficient) AND variation (e.g. standard deviation) or associated estimates of uncertainty (e.g. confidence intervals)
☐	☒	For null hypothesis testing, the test statistic (e.g. *F*, *t*, *r*) with confidence intervals, effect sizes, degrees of freedom and *P* value noted<br>*Give P values as exact values whenever suitable.*
☒	☐	For Bayesian analysis, information on the choice of priors and Markov chain Monte Carlo settings
☒	☐	For hierarchical and complex designs, identification of the appropriate level for tests and full reporting of outcomes
☒	☐	Estimates of effect sizes (e.g. Cohen's *d*, Pearson's *r*), indicating how they were calculated

Our web collection on statistics for biologists contains articles on many of the points above.

Software and code

Policy information about availability of computer code

Data collection	Custom code was used to perform recordings and obtain data.
Data analysis	Matlab R2019b, Python 3.7.6.

For manuscripts utilizing custom algorithms or software that are central to the research but not yet described in published literature, software must be made available to editors and reviewers. We strongly encourage code deposition in a community repository (e.g. GitHub). See the Nature Portfolio guidelines for submitting code & software for further information.

Data

Policy information about availability of data

All manuscripts must include a data availability statement. This statement should provide the following information, where applicable:

- Accession codes, unique identifiers, or web links for publicly available datasets
- A description of any restrictions on data availability
- For clinical datasets or third party data, please ensure that the statement adheres to our policy

Data and code will be made publicly available on Zenodo upon acceptance.

Research involving human participants, their data, or biological material

Policy information about studies with human participants or human data. See also policy information about sex, gender (identity/presentation), and sexual orientation and race, ethnicity and racism.

Reporting on sex and gender	26 females and 25 males.
Reporting on race, ethnicity, or other socially relevant groupings	Monolingual German speaking families; homogeneous socioeconomic status.
Population characteristics	Data obtained from neurotypical infants were used. Data stem from prospective longitudinal assessments between 4 and 16 weeks of post-term age.
Recruitment	Paticipants were recruited through research databases, birth registries, website advertisements, mail blasts, facebook and social media postings.
Ethics oversight	Approved by the Institutional Review Board of the Medical University of Graz, Austria (27-476ex14/15) and the University Medical Center Göttingen, Germany (20/9/19).

Note that full information on the approval of the study protocol must also be provided in the manuscript.

Field-specific reporting

Please select the one below that is the best fit for your research. If you are not sure, read the appropriate sections before making your selection.

☐ Life sciences ☒ Behavioural & social sciences ☐ Ecological, evolutionary & environmental sciences

For a reference copy of the document with all sections, see nature.com/documents/nr-reporting-summary-flat.pdf

Behavioural & social sciences study design

All studies must disclose on these points even when the disclosure is negative.

Study description	We developed a deep learning and sensor fusion approach to classify infant motor functions according to a classic diagnostic tool, the Prechtl general movement assessment (GMA). We combined three sensor modalities with fully synchronized visual, pressure and inertial movement units data to improve classification accuracy of infant movements.
Research sample	Data from a prospective longitudinal cohort of 51 (26 females) typically developing infants between 4 and 16 weeks post-term age were sampled. Data acquisition was conducted at iDN's BRAINtegrity lab at the Medical University of Graz, Austria.
Sampling strategy	Data from 45 infants from the cohort were included, who had adequate data for the required assessments administered in biweekly intervals across seven consecutive timepoints (357 datapoints assessed). We segmented the data stream into 5-second snippets. From a total of 19451 available snippets, 2800 were randomly sampled and clinically evaluated by two senior GMA assessors.
Data collection	Three fully synchronized sensor modalities were applied (visual, pressure, and inertial movement sensors) to quantify infant spontaneous motor patterns.<br>Video recordings were acquired using a standard HD camcorder with a resolution of 1920 x 1080 pixels, and 50 frames per second.<br>Pressure data was acquired using a Conformat pressure sensing mat (Tekscan, Inc., South Boston, Massachusetts, USA5. This mat contains 1024 pressure sensors arranged in a 32 x 32 array on an area of 471.4 x 471.4 mm2, producing pressure image frames (8 Bit, 32 x 32 pixels, sampling rate 100 Hz).<br>To capture infant motion using wearable sensors, six wireless Xsense MTw Awinda IMUs were applied with a sampling rate of 60 Hz. Four IMUs were each fed into a designated pocket of a customized body-suit and attached to the infant's shoulders and the outsides of the hips to assess proximal movement features. The other two IMUs were each put into a sock and attached to the soles of the infant's feet for distal features in the lower extremities.
Timing	Data collection, segmentation, annotation and analyses took place from 2017 to 2024.
Data exclusions	From the 51 participants of the cohort, data of one infant later diagnosed with a neurodevelopmental condition was excluded; another five infants were included due to incompleteness of data in the required age-periods. The final sample consists of 45 infants (23 females).
Non-participation	No particpant dropped out or declined participation
Randomization	Families with positive consent to our studies and meeting the inclusion criteria were enrolled. Participants are from a neurotypical study cohort.

Reporting for specific materials, systems and methods

We require information from authors about some types of materials, experimental systems and methods used in many studies. Here, indicate whether each material, system or method listed is relevant to your study. If you are not sure if a list item applies to your research, read the appropriate section before selecting a response.

Materials & experimental systems

n/a	Involved in the study
☒	☐ Antibodies
☒	☐ Eukaryotic cell lines
☒	☐ Palaeontology and archaeology
☒	☐ Animals and other organisms
☒	☐ Clinical data
☒	☐ Dual use research of concern
☒	☐ Plants

Methods

n/a	Involved in the study
☒	☐ ChIP-seq
☒	☐ Flow cytometry
☒	☐ MRI-based neuroimaging

Plants

Seed stocks	n/a
Novel plant genotypes	n/a
Authentication	n/a
